# Cerebral Hæmorrhage
*A Lecture delivered at the London School of Clinical Medicine.


**Published:** 1912-04-13

**Authors:** Guthrie Rankin

**Affiliations:** Physician to the "Dreadnought" and Royal Waterloo Hospitals.


### CEREBRAL HÆMORRHAGE.*

By GUTHRIE RANKIN, M.D. Glas., F.R.C.P. Lond. and Edin., Physician to the "Dreadnought" and Royal Waterloo Hospitals.

We have frequent opportunities in the wards of this hospital for investigating cases of cerebral hæmorrhage, and as one or two interesting examples of this form of disease have recently been under observation and discussion, I have thought it might be of some interest to consider the subject this afternoon from the descriptive point of view as a whole.

An apoplexy is the direct consequence of morbid changes in the cerebral circulation and is usually the result of hæmorrhage, thrombosis, or embolism. Hæmorrhage is comparatively rare before the age of forty, and is generally consequent upon degenerative changes in the walls of the arteries; thrombosis may occur at any age, and, in early adult life, is mostly a consequence of syphilis; embolism is, in the great majority of cases, secondary to mitral disease or aneurism.

### Charcot's Designation.

To-day we are concerned only with that variety of apoplexy which occurs as the result of hæmorrhage. Bleeding may occur either from a central or from a cortical vessel. It is more common from the former because the central vessels are shorter, of larger calibre, and so situated that, being called upon to sustain in greater degree than the cortical branches the shock of the ventricular systole, they are more liable to undergo degenerative changes. There is one special vessel of the anterior group of lenticulostriate arteries, somewhat larger than the others and distributed to the posterior part of the internal capsule and the neighbouring lenticular nucleus of the corpus striatum, which is so frequently the seat of rupture that it has been designated by Charcot "the artery of cerebral hæmorrhage." The effusion of blood which occurs consequent upon rupture of this vessel may take place into the substance of the corpus striatum, but more frequently extravasation occurs along the external surface of the lenticular nucleus, and, in such cases, the internal capsule being interfered with by pressure only is capable of resuming its function to a greater or less degree as the clot contracts and becomes absorbed. The hæmorrhage may be small and limited to the immediate neighbourhood of its seat of origin, but it may also be extensive enough to invade the centrum ovale or burst into the lateral ventricles, whence it may even find its way into the third, and by way of the aqueduct of Sylvius to the fourth ventricles.

Hæmorrhage may also occur into the crus, the pons, the convolutions, or the cerebellum. Meningeal hæmorrhage is not uncommon and may be due either to traumatic or non-traumatic causes.

### Intracranial Hæmorrhage.

Intracranial hæmorrhage is, in the majority of cases, the result of a diffuse periarteritis, which terminates in the production of miliary aneurisms. These aneurisms, which develop principally on the arterioles, vary in size from a millet seed to a pea, are of a reddish-brown colour, and may be either limited to a few situated in the neighbourhood of the ruptured vessel, or comprise a large number scattered throughout the area of the cerebral circulation. Though most common in advanced life when degenerative changes of all kinds are active, these small aneurisms are also met with in young subjects. Hæmorrhage also occurs as a consequence of atheromatous or fatty changes in the vessel walls, the larger arteries at the base of the brain being specially liable to this form of degeneration.

### Precedent Conditions.

The occurrence of one form or other of arterial change paves the way for hæmorrhage, but its actual occurrence is dependent upon other no less important conditions, notably upon increased tension, such as is illustratively met with in cases of gout, and cirrhosis of the kidneys; upon cerebral softening—often the result of atheromatous or syphilitic changes in the vessel walls—which, by diminishing the normal support of the vessels, renders them prone to rupture under comparatively moderate degrees of strain; and upon certain qualitative changes in the blood such as occur in scurvy, purpura, pernicious anæmia, leukæmia, and certain specific fevers.

As we are all aware, it is not at all unusual for an attack of apoplexy to occur during the night, the patient being found unconscious and stertorous in the morning, or waking to discover that he is both aphasic and hemiplegic. In many instances it may be assumed that the pathological change is a thrombosis which is encouraged by the sluggishness of the circulation through at least part of the brain during sleep. This hypothesis is not, however, sufficient to explain other cases in which an apoplexy occurring during sleep is so acute as to leave little doubt that nothing short of an extensive hæmorrhage can account for it.

### The Evidence of Dreams.

It is to be remembered that though observation has proved almost conclusively that the cortex is anæmic during sleep, it does not follow that the blood-supply of the central ganglia is likewise diminished. On the contrary, the evidence afforded by dreams, somnambulism, and other occurrences of common experience which must be dependent upon some form of cerebral activity, but which none the less occur when the consciousness of relationship to surroundings is completely under the dominion of sleep, strongly suggests that, in certain conditions not yet understood, the blood-supply of the central ganglia must be active. If

* A Lecture delivered at the London School of Clinical Medicine.

this be true, the explanation of cerebral hæmorrhage during sleep is at once evident.

### Exciting Causes.

Cerebral hæmorrhage is most liable to occur between fifty and sixty years of age, when tissue degeneration is beginning to declare itself but when the individual is still vigorous and exposed to the wear and tear of busy life. Men are attacked more frequently than women; those engaged in work involving severe muscular strain being specially liable. A hereditary tendency exists in some families to early arterial degeneration, and habits of excess in eating and drinking are an important antecedent in a large number of cases. Syphilis, gout, Bright's disease, and other disorders are, as has already been mentioned, predisposing events in many instances. Sudden effort, intense emotion, prolonged exposure to extremes of temperature, and traumatic injury are among the more frequent exciting causes of an attack.

Though the name "Apoplexy" implies a sudden and complete loss of consciousness, the onset of the attack is often gradual and may be preceded, for a longer or shorter time, by premonitory symptoms such as vertigo, headache, mental apathy, irritability of temper, numbness or formication of the limbs, etc. Nothnägel has pointed out that a sudden loss of the power of speech, regained after a few hours, is a frequent prodromal symptom.

### "A Stroke."

The symptomatology of cerebral apoplexy may be conveniently considered under two principal headings: those manifestations which belong to the actual occurrence of a "stroke," and those which belong to the consequences which arise from the local injury to the brain. The "stroke," properly speaking, is only a symptom which is best described as a complete loss of consciousness unattended by any evidence of cardiac failure or external cause to account for it. As the popular name indicates, the general impression is that the person attacked is suddenly stricken down, as though felled with an axe, but this is quite an exceptional mode of onset. Much more frequently the patient first experiences pain in the head, which is usually severe and of sudden onset, he may become sick and tumble faint into a chair or fall to the ground. He feels cold and powerless, but retains his senses, though they are dulled.

More or less rapidly he becomes drowsy and finally completely unconscious. It is in this stage, as a rule, that the doctor first sees him. He finds him with closed eyelids, flushed face, slow and stertorous breathing, a full and deliberate pulse, and a subnormal temperature. The carotids are observed to throb violently; the conjunctivæ are injected and insensitive; the pupils are usually dilated, often unequal, and sluggish in their reaction to light; the eyeballs are fixed, turned upwards, and may be conjugately deviated towards the side of the lesion; the cheeks are ballooned, one often more than the other, with each expiration; and there is frequently incontinence of both urine and fæces. Swallowing may be possible but is always difficult; and the skin is usually bathed with clammy perspiration. From this condition of profound coma the patient may never emerge. He may die after a few minutes; or he may gradually become livid, develop œdema of his lungs and succumb after some hours; or, when the power of swallowing is retained, life may be prolonged over several days in an unconscious condition, the fatal issue being preceded by increase of temperature, quickened pulse, and Cheyne-Stokes respiration.

When the hæmorrhage finds its way into the ventricle, the loss of consciousness is rapid and complete, and is sometimes accompanied by vomiting and convulsions.

### Less Severe Cases.

In cases which are less severe the initial coma gradually passes off; the pulse becomes softer and more rapid; there is a slight and temporary rise of temperature; the breathing gets less noisy; power over the sphincter returns; and, when loudly spoken to, the patient shows such signs of understanding that he may attempt to answer a simple question plainly put to him. As recovery proceeds paralysis declares itself and it is then possible from the distribution of the palsy to locate the lesion.

The loss of power can sometimes be demonstrated while the patient is still unconscious: thus on lifting the arm or leg on the affected side it may be found to be more flaccid and to drop away more dead from the grasp than on the sound side, or it may be noted that one cheek is more puffed out during expiration than the other, or there may be a manifest amount of unilateral rigidity, or the temperature may be found higher in one axilla than the other, or the plantar reflex may be extensor on the affected and flexor on the sound side.

### Temperature and Pulse.

At the onset of a hæmorrhage the temperature is usually lowered about two degrees, but it tends, in favourable cases, speedily to resume the normal level, after a temporary and moderate rise. In unfavourable cases it often suddenly rises to 104° or more within a day or two of death.

The pulse, at first full, slow, and of plus tension, becomes in favourable cases softer and less deliberate within forty-eight hours of the attack, but it always increases in rapidity in cases that do badly or terminate fatally.

The pupils, though usually dilated and irresponsive to light, are sometimes of unequal size. In pontine hæmorrhage they are often contracted to pin-points. Conjugate deviation of the eyes, accompanied by rotation in the same direction of the head, is a temporary symptom of diagnostic importance, because the deviation is always away from the paralysed side when the lesion is situated in the hemisphere, but towards it when the damage has occurred low down in the pons.

*(To be continued.)*

